# Detection of allele specific differences in *IFNL3* (*IL28B)* mRNA expression

**DOI:** 10.1186/s12881-014-0104-7

**Published:** 2014-10-05

**Authors:** Susanne Knapp, Naeem Meghjee, Sorcha Cassidy, Khaleel Jamil, Mark Thursz

**Affiliations:** Imperial College, St Mary’s Hospital, 10th floor QEQM Wing, Liver Unit, South Wharf Road, London, W2 1NY UK

**Keywords:** Variation of allele specific expression, Quantitative RT-PCR, *IFNL3* gene, Hepatitis C Virus (HCV) infection, *in vitro* assay

## Abstract

**Background:**

Variants of the interferon-lambda3 (*IFNL3*) gene have been associated with both spontaneous and treatment induced clearance of HCV infection. Attempts to link polymorphisms of the *IFNL3* gene with variation in the level of *IFNL3* expression have been inconclusive. This is partially due to the difficulty to design assays distinguishing *IFNL3* from *IFNL2*.

**Methods:**

In this study an allele specific real-time PCR (RT-PCR) assay was developed which allows the relative quantification of the two *IFNL3* transcripts in cells heterozygous for SNP *IFNL3.rs4803217* in the 3'UTR of the *IFNL3* gene. This SNP is in strong linkage disequilibrium (LD) with the predictive marker *rs12979860*.

**Results:**

Raji cells showed two-fold increased levels of *IFNL3.rs4803217* C-allele expression. In peripheral blood mononuclear cells (PBMCs) of eight uninfected donors, two donors showed increased *IFNL3.rs4803217* C-allele expression.

**Conclusion:**

This indicates that allele specific differences in *IFNL3* expression vary between individuals and might contribute to the variety of outcomes in HCV infected patients.

**Electronic supplementary material:**

The online version of this article (doi:10.1186/s12881-014-0104-7) contains supplementary material, which is available to authorized users.

## Background

Recently genetic polymorphisms in the interferon-lambda3 gene (*IFNL3*), also known as interleukin 28B (*IL-28B*) have been shown to be highly associated with the outcome and treatment response of Hepatitis C infection in ethnically diverse cohorts [1–6]. Several single nucleotide polymorphisms (SNPs) are in linkage disequilibrium (LD), which makes the identification of the functional polymorphism challenging. Attempts have been made to link the effects of the polymorphism to a difference in the level of expression of *IFNL3* transcript *in vitro* and *in vivo* [4,7] or differences in potency of the corresponding gene products [8], but no consistent difference could be detected.

The challenge with designing genotyping assays for *IFNL3* is the close homology with *IFNL2 (IL28A)*, with which it shares 96% homology on the DNA level [4,9]. Several *IFNL3* specific TaqMan assays have been designed which discriminate *IFNL3* from *IFNL2* [8,10], but are not able to discriminate between the two alleles within the gene. In order to study the allele specific expression of *IFNL3*, we developed a SYBRGreen based RT-PCR assay which is able to quantify the two allele specific transcripts of *IFNL3* (whilst excluding a signal from *IFNL2*) based around the two expressed alleles of the *rs4803217* SNP in the 3’UTR region of *IFNL3.* In the Asian and Caucasian population *SNP rs4803217* is in close LD with *rs12979860* (r^2^ = 0.98 [11,12])*,* which predicts outcome of HCV infection and treatment response. The relative amount of allele specific transcript was measured after interferon stimulation of Huh7, Raji and Jurkat cells, and in peripheral blood mononuclear cells (PBMCs) of eight uninfected healthy donors, which were heterozygous for *IFNL3.rs4803217*. The assay is a modification of allele specific quantitative RT-PCR, which has been developed by Germer *et al.* [13] to accurately measure varying allele frequencies between 5% and 95% for SNPs in pooled DNA samples. We report that the presented *IFNL3* specific assay is able to accurately measure variation of allele specific expression between individuals.

## Methods

### Cell lines

The cell lines used in our experiments were Huh7, Raji and Jurkat cell lines. The Huh7 cell line is derived from hepatocellular carcinoma [14]. The Raji cell line is a suspension cell line derived from B-lymphocytes [15]. The Jurkat suspension cell line is derived from T-cells [16]. Huh7, Jurkat, Raji cells were cultivated under standard conditions in DMEM medium containing 5% Pen/Strep and 10% heat inactivated Foetal Calf Serum (FCS) and incubated at 37°C under 5% CO2/air. Confluent Huh7 and stationary phase Raji and Jurkat cells were diluted to 2 × 10^5^ cells/ml into 1 ml of medium in 12-well plates. On the following day, when the number of cells reached 4 × 10^5^ cells/ml, cells were stimulated by adding interferon alpha (IFNα) (30–2000 Units/ml; Roferon, Roche) [17], interferon beta (IFNβ) (50–1000 Units/ml; human interferon β1a, Sigma)[18], interferon gamma (IFNγ) (50–500 ng/ul; R&D systems)[19], interferon lambda 3 (IFNλ3) (IL-28B 500 ng/ml; R&D systems) [20], toll like receptor 7 (TLR7) agonist RWJ21757 (10 μmol; R&D systems) [21] or tumour necrosis factor alpha (TNFα) (40 ng/μl; R&D systems) [22].

Huh7, Raji and Jurkat cells were tested for the expression of interleukin 10 receptor beta (*IL10RB)*, interferon alpha receptor (*IFNαR), IFNL2/3 IL28*, 2'-5'-oligoadenylate synthetase (*OAS1)* and myxovirus (influenza virus) resistance 1 (*Mx1)* (with *GAPDH* as a housekeeping gene) using primers published by Diegelmann et al. [23].

#### Peripheral Blood Mononuclear cells (PBMCs)

Peripheral blood mononuclear cells (PBMCs) from eight consented uninfected donors heterozygous for the *IFNL3.rs4803217* polymorphism were isolated using Hypaque-Ficoll (Amersham Biosciences) density centrifugation. 1–5 × 10^6^ cells in 1 ml of medium were stimulated with 800 Units /mls IFNα for 6 hours.

#### Genotyping of cell lines and healthy donors

**Figure 1:**
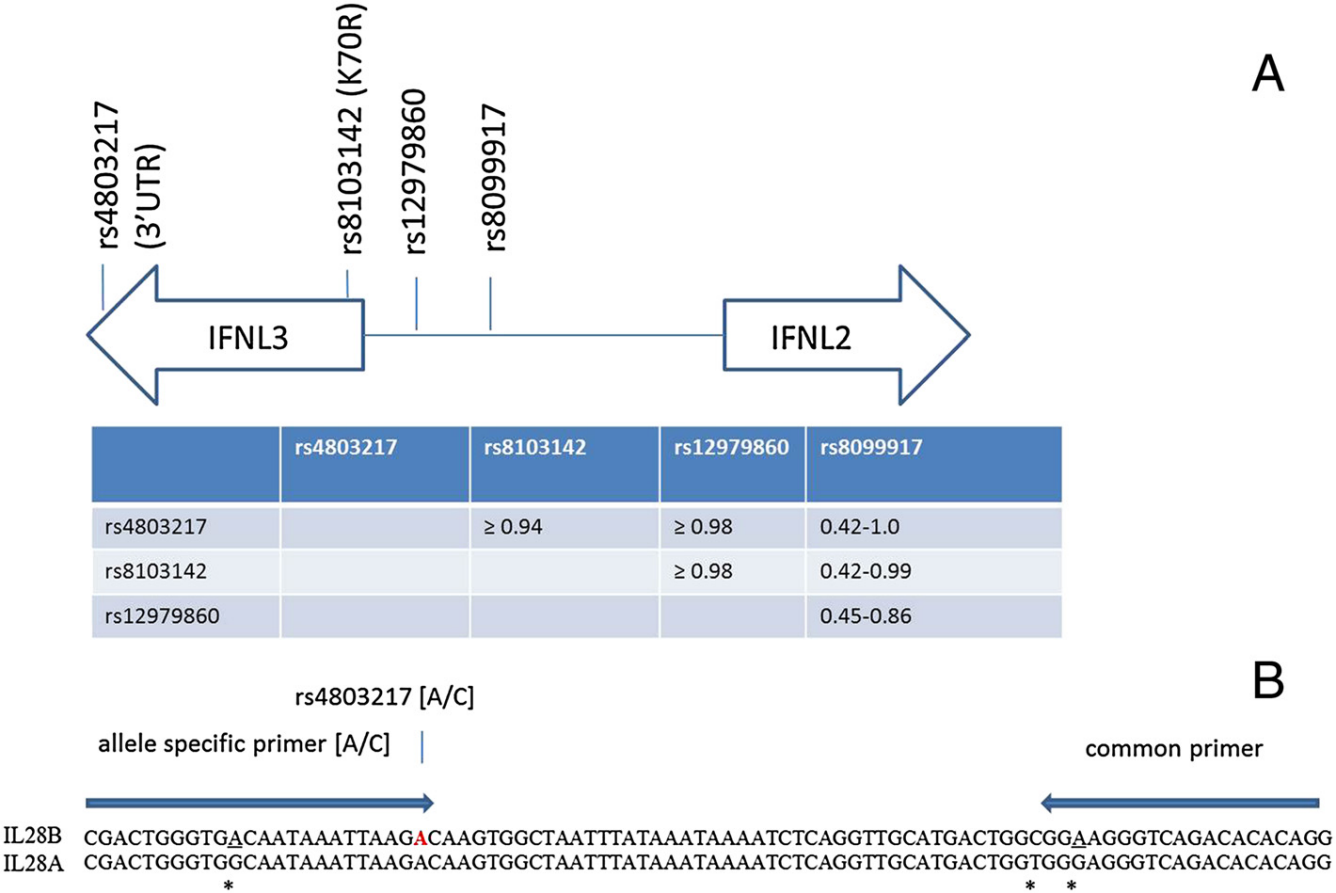
**Schematic location of the** ***rs4803217*** **SNP in relationship to other SNPs in and near the** ***IFNL2/3*** **gene**. All four variants are in high linkage disequilibrium in Caucasians (r^2^ ≥ 0.92) [12]. The linkage disequilibrium between the four markers is represented in the table below the diagram and displayed as r^2^ (10;32), with a lower linkage disequilibrium reported between *rs12979860* and *rs8099917* [11]. **(A)** The nucleotide sequence around *rs4803217* is shown for *IFNL3* and *IFNL2* with primers designed to be specific for *IFNL3* **(B)**. The *IFNL3* specific forward primer contains a mismatch to *IFNL2* at position 11 (from the 5’end) for *IFNL3* specificity. To distinguish A and C allele of the *rs4803217* SNP, the A specific primer ends with A at the 3’ end, and the C allele specific primer ends with C at the 3’ end. The reverse common primer contains one *IFNL3* specific mismatch to *IFNL2* at the 3^rd^ base from the 3’ end. Mismatches between *IFNL3* and *IFNL2* are highlighted by asterix*, and the *IFNL3* specific base within each primer is underlined.

SYBRGreen based RT-PCR was used to characterize the genomic DNA of cell lines and uninfected donors for the *IFNL3 polymorphisms rs12979860, rs8103142 and rs4803217.* The schematic location of these SNPs and their pairwise linkage disequilibrium is shown in Figure 1A. Primers were designed by the author to be specific for *IFNL3* and ordered from Invitrogen. Primer sequences are listed in Table 1. The position of the three primers for the *rs4803217* RT-PCR assay within the sequence of *IFNL2/3* is illustrated in Figure 1B.

**Table 1:** **Primer sequences for** ***IFNL3*** **genotyping and expression studies**

**SNP**	**Location**	**Allele specific primer (5’- > 3’)**	**Common primer (5’- > 3’)**
*rs12979860*	3 kb upstream	gcattcaaccctggttcA	gcttatcgcatacggctaggc
		gcattcaaccctggttcG	
*rs8103142*	exon 2 (K70R)	cttctgctgaaggactgcaA	tgagcagggctgggagg
		cttctgctgaaggactgcaG	
*rs4803217*	3’UTR	cgactgggtgacaataaattaagA	cctgtgtgtctgacccttcc
		cgactgggtgacaataaattaagC	

Each DNA sample was interrogated twice, once with the primers specific for allele 1 (A) in combination with the common primer, then with the primer specific for allele 2 (C) in combination with the same common primer. The genotyping method using the primer combination for *IFNL3.rs12979860* has been described earlier [24].

Reactions were performed in 96 well plates with 10 ng/μl genomic DNA and 0.5 μM of each primer (one allele specific and one common primer per reaction) in the presence of a SYBRGreen containing reagent mix (QuantiTect, Qiagen) using a StepOnePlus instrument (Invitrogen). The cycling conditions were initial denaturation of 10 min 95°C, 40 cycles of 15 seconds 95°C and 60 seconds 60°C, followed by a melt curve.

Genomic DNA for the validation of the method was used as 10 ng/ul after quantification with a NanoDrop spectrophotometer (Thermo Fisher).

#### cDNA synthesis

RNA was extracted from cells following the RNeasy protocol (Qiagen) and from frozen PBMCs of uninfected donors using Trizol reagent (Invitrogen) in a standard protocol including DNAse treatment. cDNA was synthesized using the Retroscript kit (Ambion) according to manufacturer’s instructions, but using oligo dTs and random decamers (2.5 μM each) in combination to optimize the efficiency of the cDNA synthesis. 1ug of RNA was transcribed into cDNA.

#### Relative quantification of gene expression by real time PCR

The same assay, including conditions and primers, which was used for genotyping was also used for relative quantification of allele specific gene expression. But in contrast to the genotyping assay, samples were run in triplicates for each allele specific reaction. 1 μl of a 20 μl of cDNA (cDNA from 50 ng of RNA) volume was put in each reaction. In order to use the assay for quantification of allele specific expression, the bias induced by the difference in amplification efficiency between the two allele specific reactions was corrected for by the use of a heterozygous genomic control in each experiment.

In order to validate the genotyping assay for its use in detecting potential 1.5 to 2 fold differences between the expression of the two alleles of *IFNL3.rs4803217*, different ratios of the two alleles from two DNAs homozygous for the respective alleles were combined and their respective dCt (difference between Ct value of allele 1 and 2) measured using the *IFNL3.rs4803217* genotyping assay. Assuming both allele specific primers have the same amplification efficiency of the assay, the reaction specific for allele1 should get amplified after the same number of amplification cycles than allele 2, and dCt should be zero for a heterozygous sample carrying the same amount of allele 1 and 2. The same results (with exception of DNA measurement and pipetting errors) should be approximated if allele 1 and allele 2 are combined in a 1:1 ratio from the two homozygous DNA samples. If the alleles are present in a ratio 1:2, with allele 2 having twice the frequency of allele 1), then allele 2 should get amplified one cycle earlier (as each cycle leads to duplication of the number of amplified copies) and the dCt should be 1. Therefore the relationship between ratio and dCt is represented by equation [1]: Ratio = 1:2^dCt^. Allele 1 (A) and 2 (C) were combined in ratios 4:1, 3.1, 2.5:1, 2:1, 1.5:1, 1:1, 1:1.5, 1:2, 1:2.5, 1:3, 1:4.

**Figure 2:**
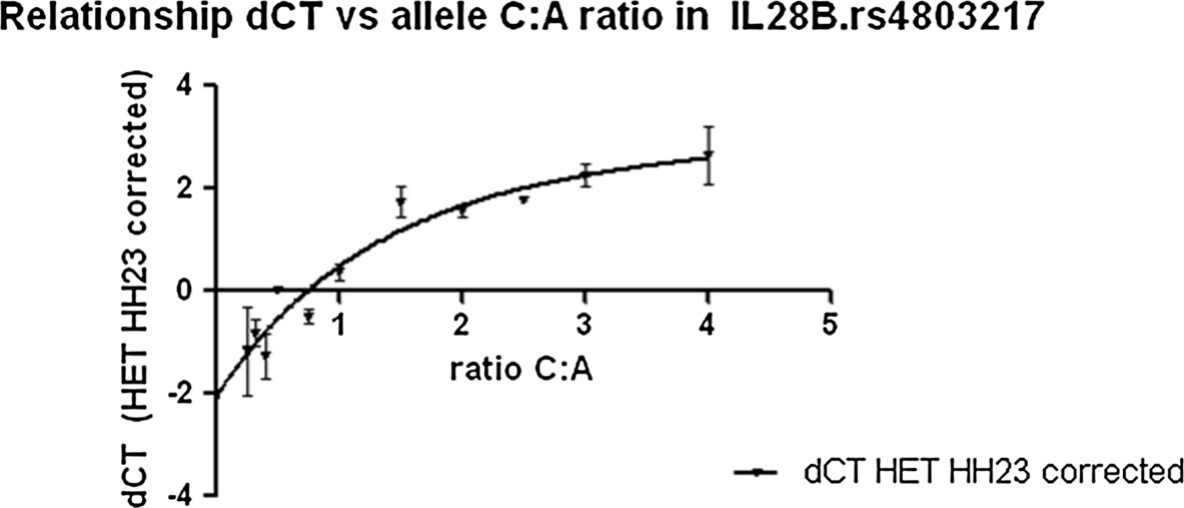
**Relationship between dCt and varying ratios of allele 1 (allele A) and 2 (allele C) measured by the** ***IFNL3.rs4803217*** **assay.** The assay shows the differences in the Ct value (dCT) of the *rs4803217* assay for different ratios of C: A allele. The differences (dCt) in the Ct value for 11 different ratios between A and C allele (4:1, 3:1, 2.5:1, 2:1, 1.5:1, 1:1, 1:1.5, 1:2, 1:2.5, 1:3, 1:4) is plotted after correction with the difference observed in the heterozygous genomic control (HH23) , where the ratio A:C is exactly 1:1. Sensitivity of *IFNL3.rs4803217* assay to detect 1.5-4 fold changes in allele specific expression. Data points are the mean of triplicate reactions and error bars indicate +/− SEM.

The results were corrected with the dCT of the heterozygote genomic DNA control (HH23), which compensates for the difference in amplification efficiency of the two alleles. The relationship between known ratios of the two alleles and measured dCt values is shown in Figure 2.

The inability of the assay to discriminate against genomic DNA present in the sample is off-set by the advantage of internal validation using three sequenced genotyping controls. As the genomic DNAs of the cells tested for variation of allele specific PCR are heterozygous for the SNP which the assay is detecting, they cannot contribute to variation in allele specific expression. Therefore any observed variation (after correction for the bias introduced through unequal amplification efficiency of the two allele specific reactions which is calculated by including a genomic heterozygous control in each assay) can be attributed to a variation in allele specific expression. RNA (without reverse transcription) controls were also included in each assay in order to detect amplification resulting from genomic DNA in the cDNA.

#### Statistical analysis

Data were analysed using Graph Pad Prism. The Mann–Whitney U test was applied to compare the means of the difference in allele expression between heterozygote genomic controls and cDNA in stimulated cells from four experiments in Raji cells, using three independently synthesized batches of cDNA from two independent stimulation events. Data obtained from the eight uninfected donors were analyzed in the same way. Two tailed p–values are calculated. Data are expressed as mean +/− SEM.

## Results

### Genotyping of cell lines and healthy donors

**Figure 3:**
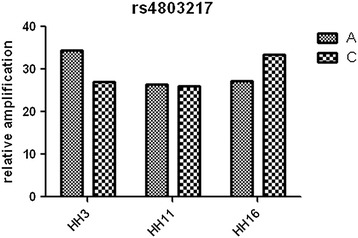
**The** ***IFNL3.rs4803217*** **assay distinguishes three** ***IFNL3*** **specific genotypes.** The Ct value is plotted for te amplification of the **A** and **C** allele specific primers respectively in each of the three genotypes.. In a sample with AA homozygocity (HH3), the A allele is getting amplified much earlier (dCt ≥ 6) in comparison to the C allele; in a sample with CC homozygocity (HH16), the C allele is getting amplified much earlier than allele A (dCT = −6). In the case of AC heterozygocity (HH11), both alleles are amplified after almost the same number of cycles (dCt = 0.06-1).

The *rs4803217* RT-PCR assay is specific for *IFNL3* (Figure 1B), as it distinguishes three genotypes (Figure 3). It excludes the amplification of *IFNL2*, which has the A allele “fixed” in the position of the *rs4803217* SNP ( 3’ end of the specific primer) . Cross reactivity with *IFNL2* would not allow the detection of CC homozygocity if this primer also recognized the A allele of *IFNL2*. The differences between the crossing points (dCt values) for the two alleles of *IFNL3.rs4803217* is robustly greater than dCT = 6 for homozygotes AA or CC in the absence of the other allele, whereas in heterozygotes the average dCt between the two allele specific reactions varies between 0.06 and 1 (assay variability) which is the result of the difference in amplification efficiency for the two alleles. To further confirm the *IFNL3* and allele specificity of the *rs4803217* assay, we sequenced amplicons which suggested AA and CC homozygocity respectively from samples which had been used as controls in each assay. We confirmed that the products were *IFNL3* and allele specific (Additional file 1: Figure S1: Sequence of amplification products).

Huh7, Jurkat and Raji cells are heterozygous for the *IFNL3.rs12979860*, *IFNL3.rs8103142*, and *IFNL3.rs4803217* polymorphisms (Figure 1A), thus making it an ideal system for the relative comparison of the expression levels of the two alleles. RNA controls revealed that only negligible amounts of genomic DNA were present in the cDNA samples used. Eight uninfected donors heterozygous for *rs4803217* were also heterozygous for *rs12979860* and *rs8103142*, which confirms the strong linkage disequilibrium between the three markers.

### Relative quantification of gene expression by real time PCR

Huh7, Raji and Jurkat cells showed constitutive expression of *IL10RB*, *IFNαR* and inducible expression of *IFNL2/3, OAS1* and *Mx1* (with *GAPDH* as a reference) after stimulation with 800 iU/ml IFNα, indicating that the receptors for the recognition of the IFN stimulation are intact and *IFNL2/3* is induced. Additional file 2: Figure S2 (induction of OAS etc after IFNa stimulation) shows the induction of *OAS* and *IFNL2/3* in Huh7 cells after 3, 6, 8, 12 and 24 hours of stimulation with 800 iU IFNα.

Initial dose response experiments demonstrated that induction of *IFNL3* expression was optimal using 800 iU/mls IFNα and 6 hour incubation (see also Additional file 2: Figure S2 (gene induction)). Therefore *IFNL3* expression for this manuscript was measured following this protocol. IFNβ showed the best induction after 6 hours of 500 iU/mls IFNβ, and IFNλ at 500 ng/ml. Both were less efficient in the induction of *IFNL3* expression and did not lead to reproducible comparisons of allele specific *IFNL3* expression. Toll like receptor 7 (TLR7) agonist RWJ21757 and tumor necrosis factor alpha (TNFα) were unable to induce detectable levels of *IFNL3* expression in Huh7 cells (data not shown) and were not tested in Raji or Jurkat cells.

As the validation experiment shows, the *IFNL3.rs4803217* allele specific PCR assay is able to measure the relationship between dCt and 1.5-2 fold changes in the ratio of the two specific alleles (Figure 2). We therefore concluded that the assay for *IFNL3.rs4803217* is suitable to measure a difference in the expression of the two alleles in cells heterozygous for the *IFNL3.rs4803217 SNP*.

**Figure 4:**
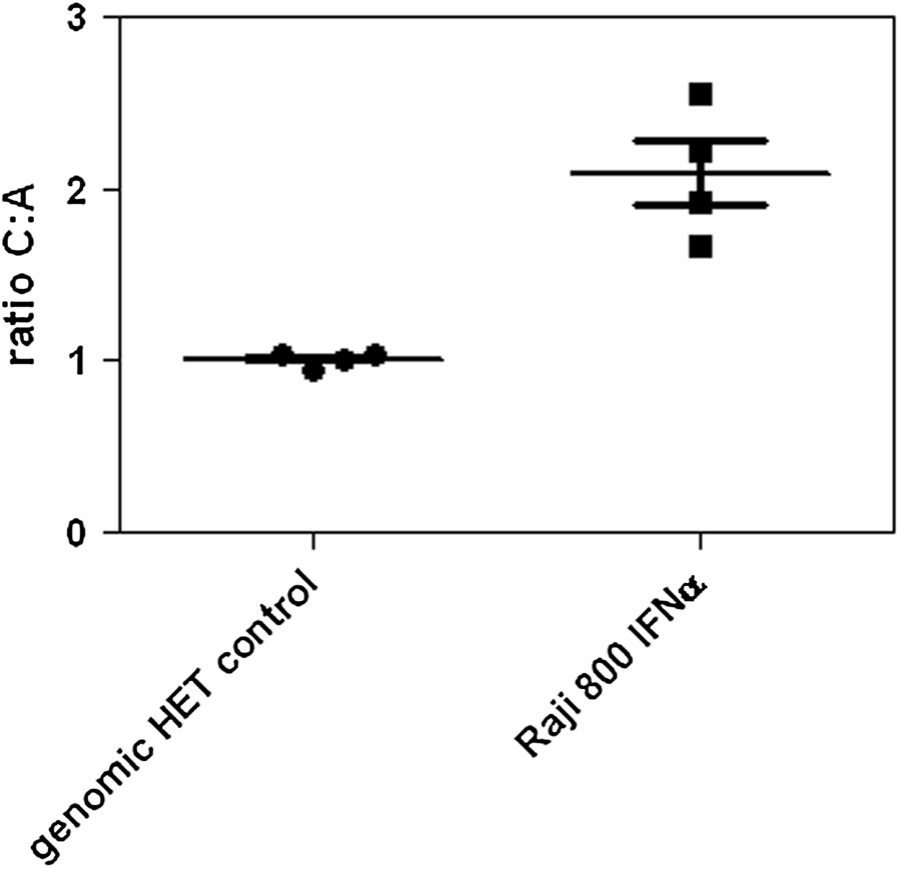
**Relative level of** ***IFNL3.rs4803217*** **C-allele specific transcript detected in Raji cells heterozygous for** ***IFNL3.rs4803217*** **after stimulation with 800 iU IFNa, compared to a genomic heterozygote control.** The ratio of C:A allele is expressed as 2^dCt after correction with the differences observed in the genomic heterozygote control. The results of 4 independent experiments are shown. The p-value 0.029 was calculated using the Mann–Whitney U test.

**Figure 5:**
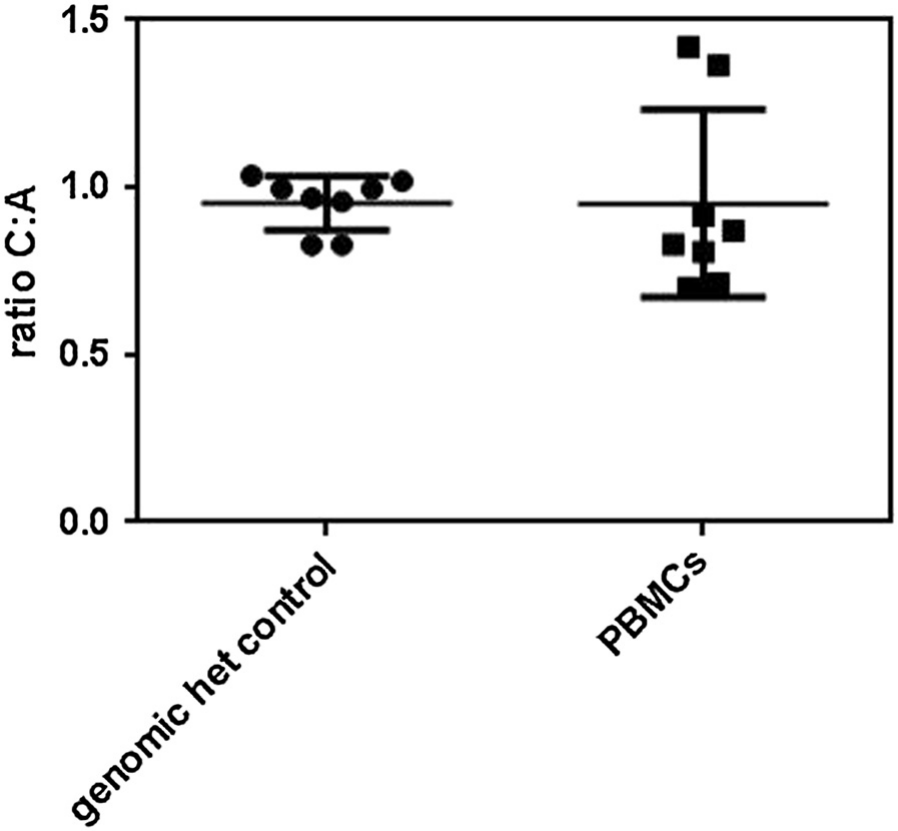
**Relative levels of** ***IFNL3.rs4803217*** **C-allele specific transcript detected in frozen PBMCs from eight uninfected donors heterozygous for** ***IFNL3.rs4803217*** **after stimulation with 800 iU IFNα, compared to a genomic heterozygote control.** The ratio of C:A allele is expressed as 2^dCt value after correction with the differences observed in the genomic heterozygous control.

Using the *IFNL3.rs4803217* genotyping based quantitative RT-PCR assay, we detected a two-fold increased expression of *IFNL3.rs4803217* C specific transcript in Raji cells in four independent experiments (fold-change 2.09 (95% CI = 0.94-1.07) versus 1.01 (95% CI = 1.50-2.70), p = 0.029). The combined result of the four experiments is represented in Figure 4. In order to validate the results in primary cells we looked at allele specific *IFNL3* expression in eight healthy donors heterozygous for the *IFNL3.rs4803217* polymorphism. The relative expression levels of the two alleles showed variation between individuals, with ratios of C to A allele ranging between 1.4 and 0.7. Two of the eight donors showed an increased *IFNL3.rs4803217* C allele expression. The results were reproducible for each individual. The differences in the ratios did not reach statistical significance (Figure 5). Due to the relatively low expression of the *IFNL3* gene in the B cells of uninfected subjects [23], we were unable to measure allele specific *IFNL3.rs4803217* expression in their B cells (data not shown)

## Discussion

In order to develop an assay that is able to measure the expressed products of *IFNL3* in an allele specific manner, the 3’UTR SNP *rs4803217* was chosen, as it is in strong linkage disequilibrium with *rs12979860,* a predictive marker for the clinical outcome of HCV infection and treatment response*.* The assay clearly excludes the amplification of *IFNL2* specific sequence as it shows the distinction of three genotypes including AA homozygocity, which would not be possible if the C allele of *IFNL2* was present. In addition our assay is also able to discriminate between the two variants of the *IFNL3.rs4803217* polymorphism. Using this assay we observe increased expression of the *IFNL3.rs4803217 C* allele in Raji cells at 2 fold level. No difference in the relative level of allele specific expression was observed for Huh7 and Jurkat cells. Raji cells are derived from a B-cell line, where high levels of IFN-λR have been detected, suggesting that B-cells are actively involved in *IFNL3* signalling [25]. Raj cells are reported to produce higher levels of *IFNL3* compared to Huh7 or Jurkat cell lines [10], which supports the finding that allele specific differences of expression can be context and tissue specific [26]. Unfortunately expression of *IFNL3* in the B-cell population of the healthy donors resulted in undetectable levels of *IFNL3* due to the low expression of *IFNL3* and smaller number of cells available (data not shown).

In order to validate our assay in primary cells, we selected eight uninfected donors heterozygous for *rs4803217* and *rs12979860.* The C allele was up-regulated in two of the eight donors. We detected marked variation in the relative ratio between the C and A allele, ranging from 1.4 to 0.7. Although this variation is not as high as the 2 fold increase as the expression of the C allele in Raji cells, and not as clear cut as a 1.5 ratio from the standard curve in Figure 2, the ratio was clearly reproducible for each donor.

The C allele of *IFNL3.rs4803217* is in LD with the *C* allele of *IFNL3.rs12979860*, which is associated with a high frequency of clearance and treatment response. Our results indicate that in certain cells or individuals the *rs12979860 CC* and also *CT* genotype are possibly producing more *IFNL3* transcript than the TT genotype, an observation which had been reported earlier using whole blood samples [4], but was not confirmed in liver tissue [8]. In contrast to Suppiah’s approach [4] where allele specific variation is measured by comparing *IFNL2/3* expression in individuals homozygous for the two alleles of *rs12979860*, our assay is specific for *IFNL3* and is carried out in heterozygotes, so that a comparison of expression between the two alleles within the same cell is less likely to be subject to confounding variables. Our results obtained in eight uninfected healthy donors heterozygous for the *IFNL3* SNP *rs4803217* (and consequently *rs12979860*) suggests that variation exists between individuals with regards to allele specific expression. As we only study the differences observed in the allele specific expression of heterozygotes, we cannot exclude that homozygosity of each of the alleles could result in a greater effect of the genotype variation.

Variation in allelic gene expression affects 20-50% of human genes and may account for variation in the transmission of diseases and disease outcomes [27–29].

It has been reported that differences in allele specific expression are inherited, but that only 3-30% of individuals exhibit the allele specific expression [27]. It was shown that allelic variation in the adenomatous polyposis coli (APC) gene expression plays a critical role in colon cancer [30]. Variation in allelic expression was also measured for the *rs2834167* SNP in *IL10RB* in Epstein Bar virus (EBV) transformed B-cell line heterozygous for this SNP and might be the underlying functional cause for the association of this SNP with the outcome of HBV infection [31].

In the literature conflicting results are reported with regards to *IFNL3* expression in PBMCs versus liver, and differences seem to exist between healthy and chronic donors. In our technical manuscript we use PBMCs of healthy donors to develop the assay as a tool which is able to measure *IFNL3* and allele specific expression of *IFNL3* transcripts in patient cohorts. A few publications have shown a potential role of blood and immune cells in the recruitment process to the liver during inflammation. Although the expression status of cells might differ between healthy donors and during the process of inflammation, PBMCs are a valid starting point for measuring allele specific expression of *IFNL3* [32].

As we did not find consistent up-regulation of the C allele in eight donors heterozygous for the C allele of *IFNL3.rs4803217* and *rs12979860*, we investigated the possibility that the observed variation in expression of the *rs4803217 C* transcript is influenced by other SNPs in the *IFNL3* region. It has been reported that a new variant upstream of the *IFNL3 gene*, which is in high linkage disequilibrium with *rs12979860,* and leads to a frameshift mutation creating a new interferon gene *IFNL4*, might explain the association with the clinical outcomes of HCV infection [33]. It has been noted that the expression of the *IFNL3* gene is dependent on the genotype of the novel *ss469415590 SNP* [34]. Genotyping all donors in our study for *ss469415590* revealed that they were all heterozygous for *ss469415590*, thus excluding the possibility of an influence of this variant.

In the three cell lines and eight donors heterozygous for *IFNL3.rs4803217* we find 100% linkage with the *IFNL3.rs12979860* marker. High LD between both markers is characteristic for Asian and Caucasian populations [12] (HapMap database r2 = 0.9 and 0.7-0.8 respectively), but reported to be at r2 = 0.4 for Sub-Saharan samples. More recently it was shown that the *IFNL3.rs4803217* variation seems to affect the stability of the transcript, which could be another way by which the *IFNL3* polymorphism exerts its function [35]. Our finding of varying C:A allele expression in certain cells and individuals could be a result of differential stability of the transcript in different individuals. Further studies need to be undertaken to explore the function of the *rs4803217* polymorphism in the outcome of HCV infection and treatment response, using an *IFNL3* and allele specific assay for *rs4803217*.

## Conclusion

In conclusion, we show that the allele specific assay for *IFNL3* which we developed is a useful tool to determine the *IFNL3.rs4803217* genotype and at the same time accurately and directly compare the expression of two alleles of the *IFNL3* gene within heterozygous cells. As the *rs4803217* SNP has been reported to have an effect on the stability of the transcript [35] the developed assay could be used for the measurement of allele specific *IFNL3* expression in patient groups with different HCV outcomes and treatment response, to determine the clinical relevance of the observed variation.

## Additional files

Additional file 1: Figure S1. Sequences of amplification products of IFNL3 allele specific *rs4803217* assay. A: The products are *IFNL3* specific at position 11 from the 5’end of the forward primer (starting from GCT; A is not part of the primer sequence), where IFNL3 is characterized by a T(A) allele, whereas *IFNL2* has a C(G) allele. The T(A) specific primer amplified the TT(AA) homozygote, whereas the C(G) specific primers amplified the GG (CC) homozygote. 5’ and 3’ describe the direction of the primer extension during the amplification. Additional file 2: Figure S2. Induction of *OAS* and *IFNL2/3* by 800iU IFNa after 3, 6, 8, 12 and 24 hours.
