# Supplementary figures and images for: Detection of allele specific differences in IFNL3 (IL28B) mRNA expression
Source: BMC Med Genet. 2014 Oct 5;15:104. doi: 10.1186/s12881-014-0104-7 (PMC4411934; doi:10.1186/s12881-014-0104-7)

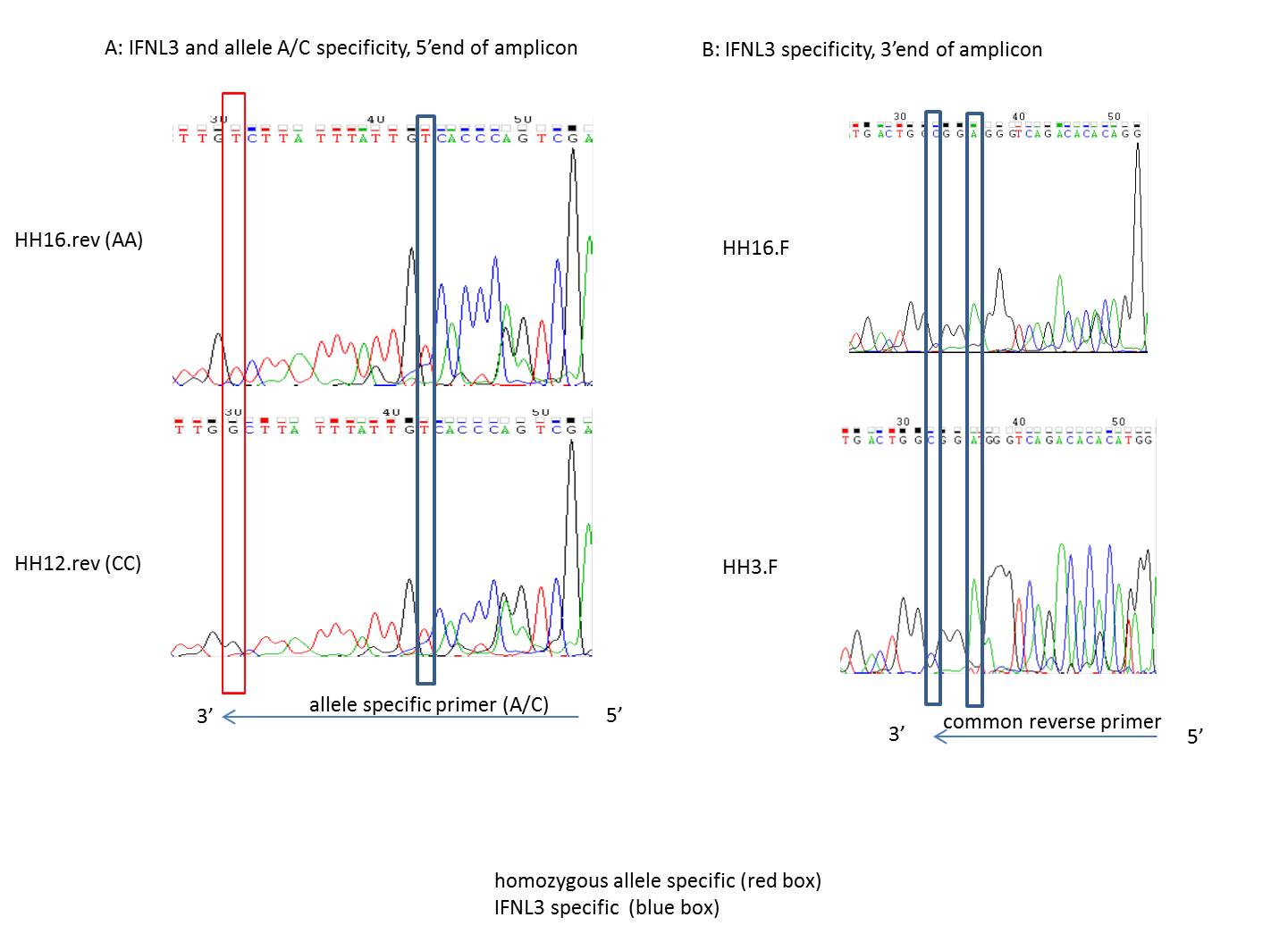

Supplement: Additional file 1: Figure S1. — Sequences of amplification products of IFNL3 allele specific rs4803217 assay. A: The products are IFNL3 specific at position 11 from the 5’end of the forward primer (starting from GCT; A is not part of the primer sequence), where IFNL3 is characterized by a T(A) allele, whereas IFNL2 has a C(G) allele. The T(A) specific primer amplified the TT(AA) homozygote, whereas the C(G) specific primers amplified the GG (CC) homozygote. 5’ and 3’ describe the direction of the primer extension during the amplification. [file 12881_2014_104_MOESM1_ESM.jpeg]

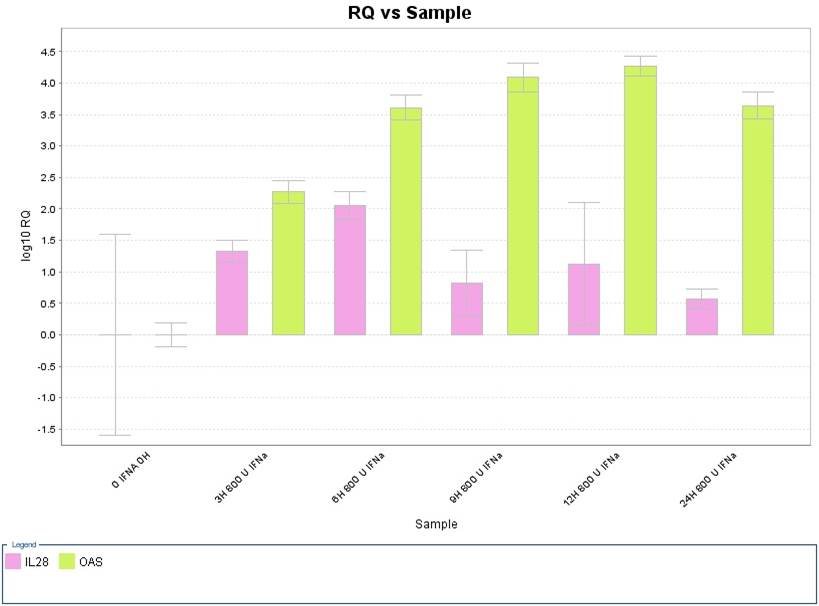

Supplement: Additional file 2: Figure S2. — Induction of OAS and IFNL2/3 by 800iU IFNa after 3, 6, 8, 12 and 24 hours. [file 12881_2014_104_MOESM2_ESM.jpeg]
